# Palmatine, a Bioactive Protoberberine Alkaloid Isolated from *Berberis cretica*, Inhibits the Growth of Human Estrogen Receptor-Positive Breast Cancer Cells and Acts Synergistically and Additively with Doxorubicin

**DOI:** 10.3390/molecules26206253

**Published:** 2021-10-15

**Authors:** Aneta Grabarska, Paula Wróblewska-Łuczka, Wirginia Kukula-Koch, Jarogniew J. Łuszczki, Eleftherios Kalpoutzakis, Grzegorz Adamczuk, Alexios Leandros Skaltsounis, Andrzej Stepulak

**Affiliations:** 1 Department of Biochemistry and Molecular Biology, Medical University of Lublin, Chodzki 1, 20-093 Lublin, Poland; andrzejstepulak@umlub.pl; 2 Department of Pathophysiology, Medical University of Lublin, Jaczewskiego 8b, 20-090 Lublin, Poland; paula.wroblewska-luczka@umlub.pl (P.W.-Ł.); jarogniew.luszczki@umlub.pl (J.J.Ł.); 3 Department of Pharmacognosy with Medicinal Plants Garden, Medical University of Lublin, Chodzki 1, 20-093 Lublin, Poland; virginia.kukula@gmail.com; 4 Laboratory of Pharmacognosy and Natural Products Chemistry, School of Pharmacy, National and Kapodistrian University of Athens, Panepistimioupoli Zografou, 15771 Athens, Greece; elkalp@pharm.uoa.gr (E.K.); skaltsounis@pharm.uoa.gr (A.L.S.); 5 Independent Medical Biology Unit, Medical University of Lublin, Jaczewskiego 8b, 20-090 Lublin, Poland; grzegorzadamczuk@umlub.pl

**Keywords:** palmatine, isoquinoline alkaloids, *Berberis cretica*, Berberidaceae, breast cancer, isobolographic analysis, natural products, HPLC-MS

## Abstract

Palmatine (PLT) is a natural isoquinoline alkaloid that belongs to the class of protoberberines and exhibits a wide spectrum of pharmacological and biological properties, including anti-cancer activity. The aim of our study was to isolate PLT from the roots of *Berberis cretica* and investigate its cytotoxic and anti-proliferative effects in vitro alone and in combination with doxorubicine (DOX) using human ER^+^/HER2^−^ breast cancer cell lines. The alkaloid was purified by column chromatography filled with silica gel NP and Sephadex LH-20 resin developed in the mixture of methanol: water (50:50 *v*/*v*) that provided high-purity alkaloid for bioactivity studies. The purity of the alkaloid was confirmed by high resolution mass measurement and MS/MS fragmentation analysis in the HPLC-ESI-QTOF-MS/MS-based analysis. It was found that PLT treatment inhibited the viability and proliferation of breast cancer cells in a dose-dependent manner as demonstrated by MTT and BrdU assays. PLT showed a quite similar growth inhibition on breast cancer cells with IC_50_ values ranging from 5.126 to 5.805 µg/mL. In contrast, growth of normal human breast epithelial cells was not affected by PLT. The growth inhibitory activity of PLT was related to the induction of apoptosis, as determined by Annexin V/PI staining. Moreover, PLT sensitized breast cancer cells to DOX. Isobolographic analysis revealed synergistic and additive interactions between studied agents. Our studies suggest that PLT can be a potential candidate agent for preventing and treating breast cancer.

## 1. Introduction

Breast cancer (BC) is currently one of the most frequently diagnosed cancersin the world and the leading cause of cancer death, followed by colorectal and lung cancer [1]. BC is a complex, genetically and clinically heterogenous disease with multiple subtypes. According to the World Health Organization (WHO) classification, there are about 20 distinct histological types of BC [2], the largest percentage of which are invasive ductal carcinoma (IDC) or invasive lobular carcinoma (ILC) [3]. In addition, the introduction of advanced methods of molecular analysis such the next-generation sequencing and related strategies has made it possible to developa newer breast cancer classification system based on tumor biology [4]. In clinical practice, molecular subtypes of breast cancer are classified based on prognostic and predictive markers, including steroid hormone receptor status such as estrogen receptor (ER) and progesterone receptor (PR) and the presence or absence of the human epidermal growth factor receptor 2 (HER2), also known as ErbB-2, ERBB2, or HER2/neu [5]. Breast cancers are divided into luminal A (ER^+^/PR^+^/HER2^−^) and HER2-enriched and triple-negative (TNBC) which is negative for all three receptors. The HER2^+^ subtype can be further categorized into luminal B (ER^+^/PR^+^/HER2^+^) and ER^−^/PR^−^/HER2^+^ [6]. Prognosis and chance for survival varies by stage and genotype of breast cancer. Early-stage breast cancers have a lower risk of recurrence and a more favorable prognosis with a five-year survival rate of 99%. Meanwhile, the five-year relative survival of high-stage tumors is 25% [7]. Moreover, compared with luminal subtypes breast cancers, TNBC and HER2-overexpessed patients have worse clinical outcomes [8].

At present, numerous treatment strategies are available for BC, including surgery, chemotherapy, radiotherapy and/or hormonal therapy based on selective estrogen receptor modulators (tamoxifen and fulvestrant), and aromatase inhibitors [9]. The HER2 positive patients can be treated with targeted therapy with monoclonal antibodiessuch as trastuzumab and pertuzumab [10]. Doxorubicin (DOX or Adriamycin—the commercial name), is an anthracycline drug used to treat both hematologic malignancies and numerous solid tumors, including breast cancer [11]. DOX can exert its anti-cancer activities by intercalating and interfering with DNA and RNA synthesis [12]. DOX acts as an inhibitor of topoisomerase II as well as triggering DNA damage through free radicals production [13]. Standard chemotherapy containing both an anthracycline and a taxane is generally recommended for patients with TNBC, HER2-positive breast cancers, and high-risk luminal tumors [14]. Clinical application of DOX is limited by a dose-related risk of cardiotoxicity that may progress toward dilated cardiomyopathy and systolic heart failure [15]. An important cause of therapeutic failure of breast cancer treatment is developing drug resistance [16]. Therefore, it is important to search for new substances that have both anti-cancer, and chemopreventive properties and that support oncological treatment. In the last years, phytochemicals such as flavonoids, alkaloids, polysaccharides, essential oils, quinonoids, terpenoids, coumarins and saponins found in edible and non-edible plants have attracted particular interest because of their broad spectrum of biological activity [17].

**Figure 1:**
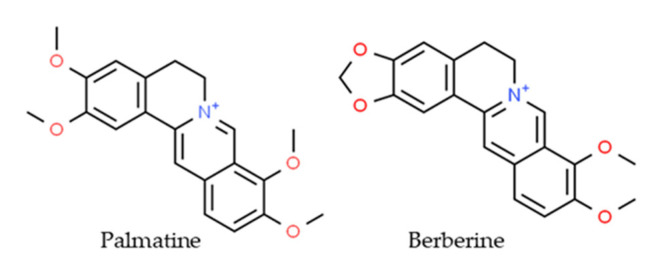
The chemical structure of palmatine and berberine.

Palmatine (PLT) is a bright yellow compound—a natural isoquinoline alkaloid belonging to the class of protoberberines found in Berberidaceae, Papaveraceae, Ranunculaceae, and Menispermaceae botanical families [18]. PLT is structurally similar to a well-known alkaloid berberine with slight differences in the substitution on the isoquinoline moiety (Figure 1) [19].

Both palmatine and berberine are quaternary ammonium salts. The former is substituted with four methoxyl groups contrary to berberine which contains a methylenedioxy moiety at C2 and C3 of the tetracyclic structure [20,21]. PLT has a wide spectrum of pharmacological and biological activities, including anti-inflammatory, antiviral, and neuroprotective properties [22], and it has been used in the treatment of jaundice, dysentery, hypertension, inflammation, and liver-related diseases [23]. PLT and its derivatives have also been shown to exhibit significant antitumor activity against prostate cell lines, with DU145 being the most sensitive [24]; hepatocellular cell lines such as QGY-7701, SMMC-7721, and HepG2; lymphoblastic cells originally derived from a child with acute lymphoblastic leukemia (CEM), Lewis lung carcinoma, breast cancer cell line (MCF-7); cells derived from a malignant glioblastoma tumor (U251); colon cancer cell line (HT-29); and cervical cancer cell line (SiHa) [25].

The aim of our study was to isolate palmatine from the roots of *Berberis cretica* and investigate its cytotoxic and anti-proliferative effects in vitro alone and in combination with DOX in three human ER^+^/HER2^−^ breast cancer cell lines. The detailed antiproliferative interactions between PLT and DOX were analyzed by the isobolographic analysis, which assesses the presence of synergy, additive effects or antagonism.

## 2. Results

### 2.1. The Isolation of Palmatine from Berberis cretica Methanolic Root Extract by Column Chromatography

**Figure 2:**
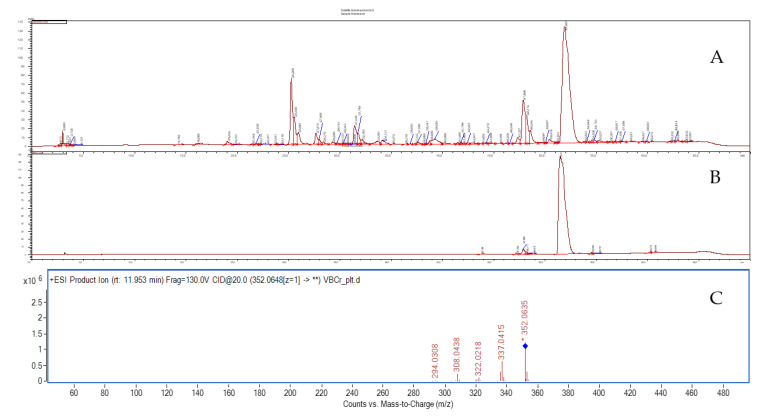
The HPLC-DAD chromatogram of the total extract from Berberis cretica root recorded at 280 nm (**A**); the HPLC-DAD chromatogram of purified palmatine(**B**); the MS/MS spectrum of palmatine recorded in the fragmentation energy of 130 V and collision energy of 20V (**C**).

Our previous studies of the extracts from the roots of *Berberis cretica* led to the identification of several isoquinoline alkaloids including [26,27] magnoflorine, berberine, palmatine, jatrorrhizine, berbamine and others. Palmatine was obtained from the methanolic extract using a traditional chromatographic technique—namely medium pressure liquid chromatography. The compound was isolated on a Sephadex column with a polar solvent system (methanol/water 50:50 *v*/*v*) at a purity of 94.5%. A simple composition of the obtained extract allowed for the effective application of this traditional chromatographic technique to isolate high-purity palmatine. The identity of alkaloid was confirmed in the HPLC-MS analysis and was based on high resolution mass measurement and on the analysis of the fragmentation pattern of the molecular ion. Palmatine was identified in the spectra recorded in the positive ionization mode, with the fragmentation energy setting of 130 V and in the collision energy of 20 V using the HPLC-ESI-QTOF-MS/MS platform. Under these conditions, the compound of interest delivered the following *m*/*z* fragments: 337, 322, 308, and 294 u. The signals corresponded to the subsequent detachments of methyl groups from the molecular ion as described earlier [28]. Specifically, the *m*/*z* signal of 337 was derived from the loss of a methyl group of [M-CH_3_]^+^, while the 322, 308, and 294 u signals resulted from following detachments of one, two, and three further methyl groups from the molecular ion, respectively (Figure 2). Although the applied voltage did not allow for the decomposition of the isoquinoline ring system, the obtained data together with high resolution mass measurement values of the parent ion and its fragments provided sufficient data for the tentative identification of palmatine in the fractions of *Berberis cretica* root extract.

### 2.2. The Cytotoxic and Anti-Proliferative Effect of PLT on the Human Breast Cancer Cell Lines

**Figure 3:**
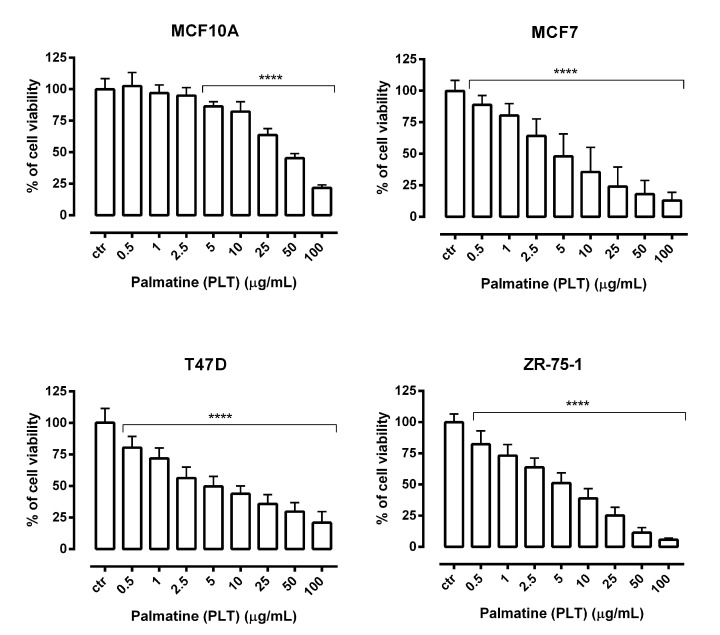
The effect of palmatine (PLT) on the viability of human breast cancer cell lines was measured by MTT assay after 72 h. Results are presented as mean ±SEM at each concentration. (**** *p* < 0.0001), n = 40 per concentration from five independent experiments.

Growth inhibition studies are typically the first step in determining a potential anticancer activity of new compounds. Therefore, the cell growth inhibitory activity of PLT was evaluated using MTT and LDH assays, based on mitochondrial enzyme activity and cell membrane permeability, respectively. In present study, MCF7, T47D, and ZR-75-1 breast cancer-derived cell lines or MCF10A normal human breast epithelial cells were treated with various concentrations of PLT. As shown in Figure 3, PLT reduced the viability of studied breast cancer cells in a dose-dependent manner as compared with control (untreated cells). In contrast, MCF10A cells were comparatively resistant to PLT treatment. Statistically significant inhibition of the viability of normal cells was observed in the concentration range of 5–100 µg/mL of PLT, whereas all studied breast cancer cells were sensitive to the lowest concentration of PLT (0.5 µg/mL).

**Figure 4:**
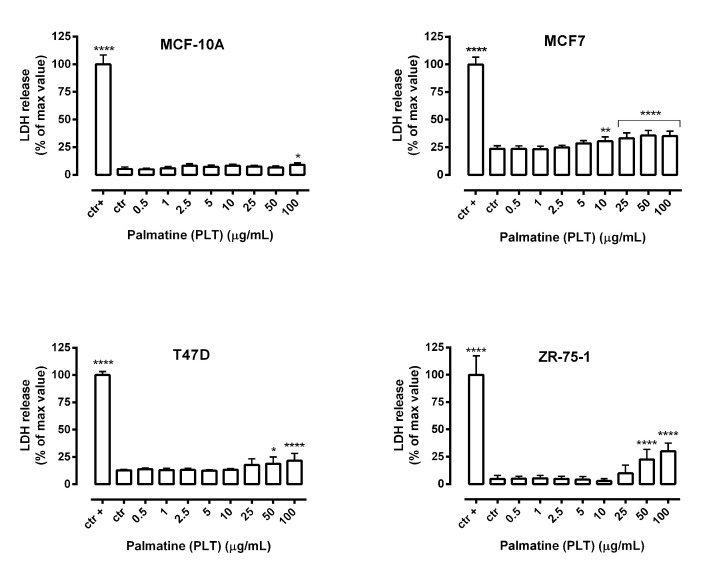
Cytotoxicity of palmatine (PLT) to normal human breast epithelial cells (MCF-10A) and breast cancer cells (MCF7, T47D, and ZR-75-1). Lactate dehydrogenase ELISA kit was used to quantify cytotoxicity by measuring LDH activity released from damaged cells. Normal breast epithelial cells and breast cancer cells were incubated for 72 h alone or in the presence of PLT (0.5–100 µg/mL). The results are presented as the percentage in LDH release to the medium by treated cells vs. cells grown in control medium (ctr) and cells treated with lysis buffer (ctr+). Data are presented as mean ±SEM at each concentration. * *p* < 0.05; ** *p* < 0.01 and **** *p* < 0,0001 vs. control group (Tukey’s test), n = 24 from three independent experiments.

The release of cytoplasmic enzyme LDH into the culture medium due to cell membrane damage is a measure of irreversible cell death and is a one feature of both necrotic cells and late apoptosis [29]. In our study, a significant increasein LDH leakage was observed in MCF7 cells treated with PLT at the concentration range of 10–100 µg/mL. Importantly, the percentage of LDH released from T47D and ZR-75-1 cells remained unaffected at concentrations of PLT below 50 µg/mL. It is noteworthy that the cytotoxic effect of PLT on normal human breast epithelial cells MCF-10A was lower compared withtested breast cancer cells and at the high concentration range (Figure 4). Furthermore, the final concentration of dimethyl sulfoxide (DMSO) in the culture medium, used to dissolve PLT, did not exceed 0.1% and did not affect cell viability and cell membrane integrity (data not shown).

**Figure 5:**
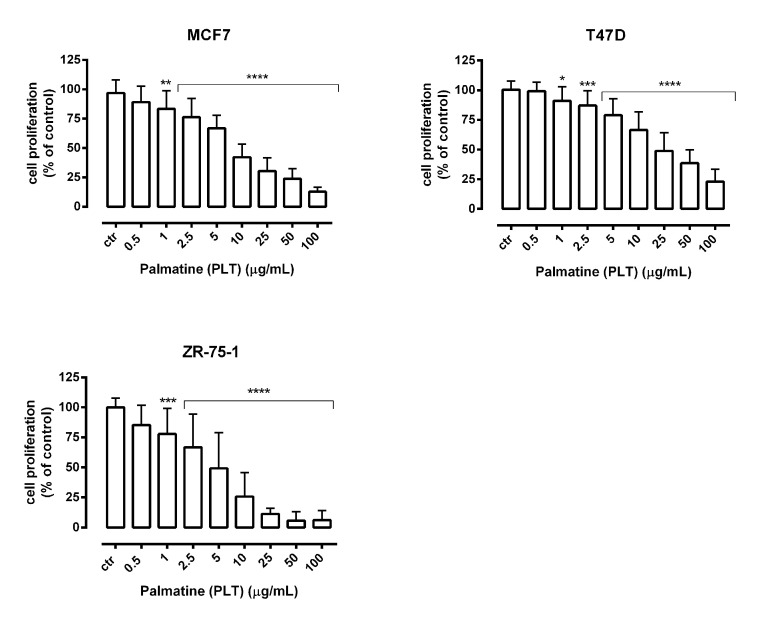
The effect of palmatine (PLT) on the proliferation of human breast cancer cell lines was measured by BrdU assay after 72 h. Results are presented as mean ±SEM at each concentration. (* *p* < 0.05; ** *p* < 0.01; *** *p* < 0.001, **** *p* < 0.0001), n = 16 per concentration from two independent experiments.

Additionally, PLT administered alone dose-dependently reduced the proliferation of MCF7, T47D, and ZR-75-1 breast cancer cells as evaluated by measuring BrdU (5-bromo-2′-deoxyuridine) incorporation into cellular DNA in proliferating cells (Figure 5).

### 2.3. The Treatment of Breast Cancer Cells with PLT Resulted in a Significant Increase in Apoptotic Cell Population

**Figure 6:**
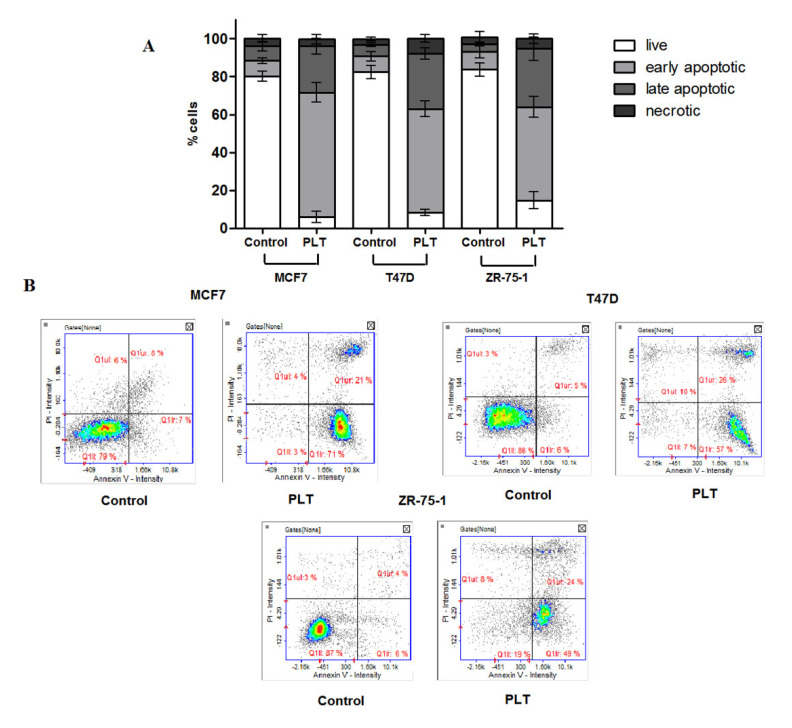
(**A**) Detection of cell apoptosis/necrosis in MCF7, T47D and ZR-75-1 cells with Annexin V-FITC and propidium iodide staining using image cytometry. The cells were treated for 72 h at the IC_50_-specific PLT concentration. Values were presented as mean ±SD derived from three independent experiments. (**B**) The results show one representative experiment of three independently performed. Q1II-live, Q1lr- early apoptotic, Q1ur- late apoptotic, and Q1uI- necrotic cells.

Next, to determine whether apoptosis is involved in the cytotoxic effect of PLT, the translocation of phospholipid phosphatidyloserine (SE) to the outer plasma membrane was measured using annexin V/propidium iodide (PI) staining. It is well known that the loss of phospholipid asymmetry, leading to the externalization of SE, is one of the features of apoptotic cell death. Cells with exposed SE are recognized by Annexin V, a calcium-dependent phospholipid-binding protein. Moreover, the combination of Annexin V and PI allows for the distinction between early apoptotic cells (Annexin V^+^/PI^−^), late apoptotic cells (Annexin V^+^/PI^+^), and necrotic cells (Annexin V^−^/PI^+^). In our studies, the treatment of breast cancer cells with PLT resulted in a strong increase in the percentage of cells in early apoptosis. The most remarkable effect was obtained in the case of the MCF cell line (at least a 70% increase). On the other hand, the application of PLT led to early apoptosis in approximately 50% of cells in both the T47D and ZR-75-1 cell lines. We also observed a moderate increase in the number of Annexin V/PI positive cells (at a level of 20% of cells). Necrosis remained at a level lower than 10% (Figure 6).

### 2.4. Effects of PLT in Combination with DOX on the Viability of Breast Cancer Cells

**Figure 7:**
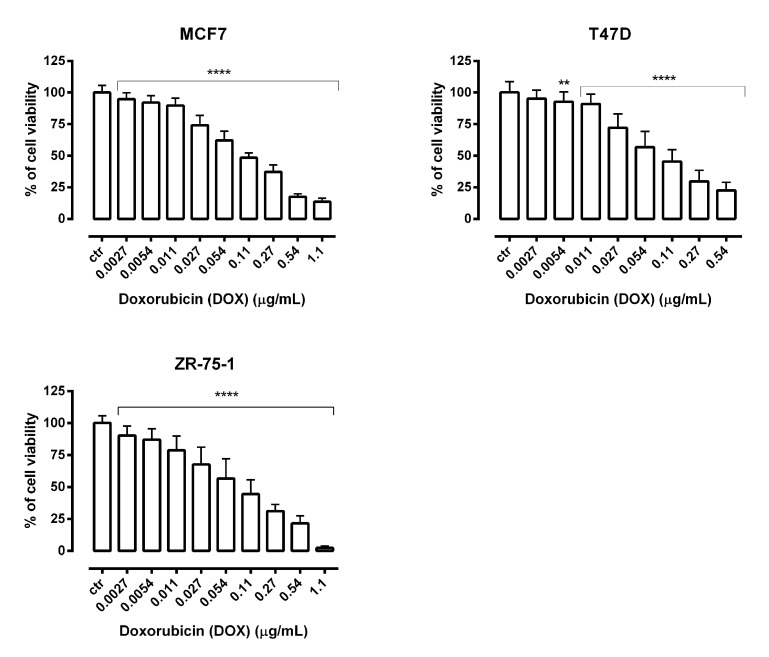
The effect of doxorubicin (DOX) on the viability of human breast cancer cell lines was measured by MTT assay after 72 h. Results are presented as mean ±SEM at each concentration. (** *p* < 0.01; **** *p* < 0.0001), n = 40 per concentration from five independent experiments.

We analyzed the concentration-related inhibitory effects of DOX on breast cancer cells to subsequently find out whether its combination with PLT can enhance anticancer activity of this chemotherapeutic. Our studies showed that DOX administered alone markedly inhibited the viability of MCF7, T47D and ZR-75-1 breast cancer cells and the results are presented in Figure 7.

**Figure 8:**
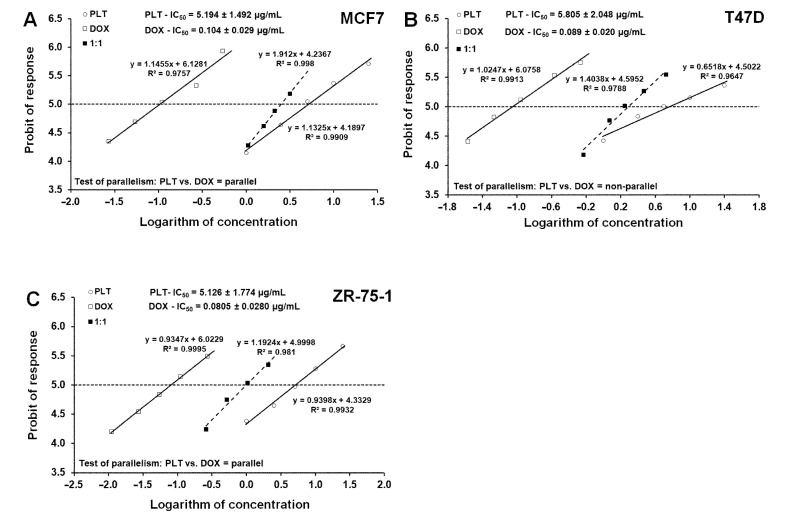
(**A**–**C**) Log-probit dose–response relationship curves (DRRCs) for palmatine (PLT) and doxorubicin (DOX) administered alone, and in combinations at the fixed ratio of 1:1 (dotted line), illustrating the anti-proliferative effects of the drugs in the human breast cancer cell lines (MCF7, T47D, and ZR-75-1) measured in vitro by the MTT assay. Doses of PLT and DOX administered separately and the mixture of the drugs at the fixed-ratio combination of 1:1 (dotted line) were transformed into logarithms, whereas the anti-proliferative effects produced by the drugs in the human breast cancer cell lines (MCF7, T47D, and ZR-75-1) measured in vitro by the MTT assay were transformed into probits according to Litchfield and Wilcoxon (1949). Linear regression equations of DRRCs are presented on the graph; where *y* is the probit of response, and x is the logarithm (to the base 10) of a drug dose, and R^2^ is the coefficient of determination. Test for parallelism revealed that the experimentally determined DRRCs for PLT and DOX (administered alone) are not parallel to one another in T47D cancer cell line and are parallel to one another in MCF7 and ZR-75-1 cancer cell lines.

Next, log-probit linear regression analysis described by Litchfield and Wilcoxon [30] was performed. The median inhibitory concentrations (IC_50_ values) were calculated from equations of log-probit dose–response relationship curves (DRRCs) for PLT and DOX (Figure 8A–C) and are summarized in Table 1. We observed that both PLT and DOX showed quite a similar antiproliferative effect on the breast cancer cells, with IC_50_ values ranging from 5.126 to 5.805 µg/mL and from 0.080 to 0.104 µg/mL, respectively. The test for parallelism of DRRCs between DOX and PLT revealed that the DRRCs of both compounds were non-parallel to each other in the T47D cell line and parallel in both the MCF7 and ZR-75-1. We also found that co-administration of PLT and DOX enhanced drug-mediated cytotoxicity. Compared with the IC_50_ concentration of DOX alone, the anti-proliferative effects were the same or even better using ½ IC_50_ concentration of both DOX and PLT. After the combination of ½ IC_50_ PLT and ½ IC_50_ DOX, the percentage of dead cells was 53.21% (MCF7), 60.48% (T47D), and 68.06% (ZR-75-1) (data not shown). These findings clearly indicate that concurrent administration of both agents seems to be justified.

### 2.5. Isobolographic Analysis of Interaction between DOX and PLT in Human Breast Cancer Cell Lines

**Figure 9:**
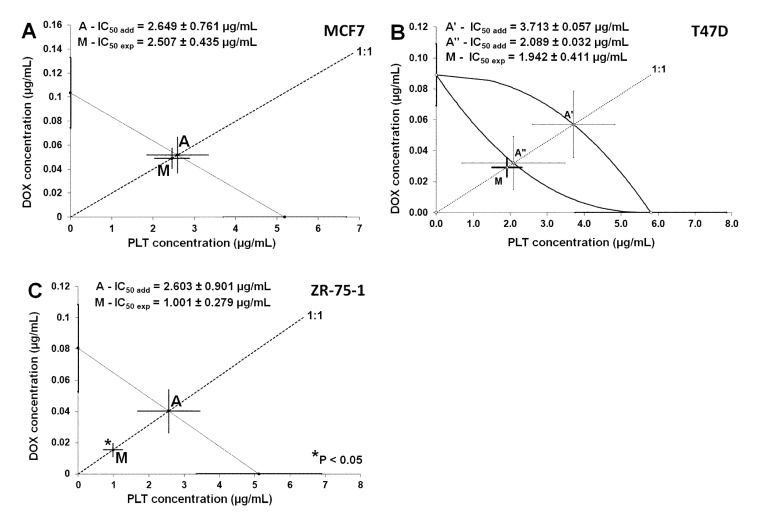
Isobolograms showing additive (**A**,**B**) and synergistic (**C**) interactions between doxorubicin (DOX) and palmatine (PLT) with respect to their anti-proliferative effects on MCF7 (**A**), T47D (**B**) and ZR-75-1 (**C**) breast cancer cell lines measured in vitro by the MTT assay. The median inhibitory concentrations (IC_50_) for DOX and PLT are plotted graphically on the *x* and *y* axes, respectively. The solid lines on the *x* and *y* axes represent the SEM for the IC_50_ values of the studied drugs administered alone. The curves connecting the IC_50_ values for PLT and DOX administered alone represent the lower and upper isoboles of additivity. The dotted line starting from the point (0,0) corresponds to the fixed-ratio of 1:1 for the combination of DOX with PLT. The diagonal line connects the IC_50_ for DOX and PLT on the *x* and *y* axes. The points A’ and A” depict the theoretically calculated IC_50add_ values for both, the lower and upper isoboles of additivity. The experimentallyderived IC_50exp_ value (point M) for total dose of the mixture expressed as the proportion of DOX and PLT that produced a 50% antiproliferative effect in breast cancer cell lines measured in vitro by the MTT assay. On the graph, the SEM values are presented as horizontal and vertical error bars for every IC_50_ value.

The isobolographic analysis of interaction for non-parallel DRRCs revealed that the mixture of DOX with PLT at the fixed-ratio of 1:1 exerted additive interaction on T47D cells (Figure 9B). The experimentally derived IC_50exp_ value for this fixed-ratio combination was 1.942 ± 0.411 μg/mL, whereas the additively calculated IC_50add_ values were 2.089 ± 0.032 μg/mL (for the lower line of additivity) and 3.713 ± 0.0587 μg/mL (for the upper line of additivity), respectively. Similarly, the mixture of DOX with PLT in MCF7 cells also exerted additivity. The experimentally derived IC_50 exp_ value for the mixture of DOX with PLT in MCF7 cells was 2.507 ± 0.435 μg/mL. The isobolographic analysis of interaction for parallel DRRCs did not reveal any significant differences between the IC_50exp_ and IC_50add_ values with unpaired Student’s *t*-test in the MCF7 cancer cell line (Figure 9A). In contrast, the isobolographic analysis of interaction for parallel DRRCs revealed that the mixture of DOX with PLT at the fixed-ratio of 1:1 exerted a synergistic interaction in ZR-75-1 cells. The experimentally derived IC_50exp_ value for this fixed-ratio combination was 1.001 ± 0.279 μg/mL, whereas the additively calculated IC_50add_ value were 2.603 ± 0.901 μg/mL. Thus, the IC_50exp_ value significantly differed (* *p* < 0.05) from the IC_50add_ values with the Student’s *t*-test (Figure 9C).

## 3. Discussion

Cancer is the second leading cause of death worldwide. Lung, prostate, colorectal, stomach and liver cancers are the most common types of cancer in men, while breast, colorectal, lung, cervical, and thyroid cancers are the most common among women [31]. Despite the rapid progress in adjusting the treatment to the specific biological characteristics of cancer and the development of new anticancer strategies that are more modern, less burdensome, and less negatively impacting on the quality of life, breast cancer is still a progressive disease in many patients [32]. Intensive research explores novel breast cancer therapeutics with the focus on clinical application. The numerous studies conducted in the last decade have shown that some of the herbal ingredients—called secondary metabolites and including, e.g., curcumin, epigallocatechin gallate, berberine, artemisinins, ginsenosides, ursolic acid, silibinin, emodin, triptolide, cucurbitacins, tanshinones, ordonin, shikonin, gambogic acid, artesunate, wogonin, β-elemene, and cepharanthine—are of great importance in preventing and treating many diseases, including cancer [33,34,35,36]. It is noteworthy that many of the used anti-cancer therapeutics originate from natural sources. Some of the examples that may be listed at this point include irinotecan, vincristine, etoposide, and paclitaxel from plants; actinomycin D and mitomycin C from bacteria; and a marine-derived bleomycin [37]. Natural products are often treated as scaffolds for the following semisynthesis of active anticancer drugs. That is why it is of high importance to perform screening on single secondary metabolites to select new drug candidates with promising pharmacological profiles.

*Berberis cretica* is a thorny shrub of the Berberidaceae family that is widely spread around the Mediterranean Sea basin [38]. Generally, fruits, leaves, bark, and roots of *Berberis* species have been reported for their wide range of pharmacological activities, including tonic, antimicrobial, antiemetic, antipyretic, antipruritic, antioxidant, antiinflammatory, hypotensive, antiarrhythmic, sedative, antinociceptive, anticholinergic and cholagogic properties. In folk and traditional medicine, *Berberis* species have been used to treat various types of diseases, such as cholecystitis, cholelithiasis, jaundice, dysentery, leishmaniasis, malaria, gall stones, hypertension, ischemic heart diseases, cardiac arrhythmias and cardiomyopathies [39]. While palmatine has been listed as the major constituent of *Berberis cretica* roots, many other alkaloids that belong to isoquinolines were also identified in barberry shrubs, such as berberine, magnoflorine, jatrorrhizine, oblongine, karakoramine, oxyacanthine, berbamine, penduline, thalrugosine, isotetrandrine, obaberine, isoboldine, glaucine, berberubine, oxyberberine and others [27,40,41]. Nonetheless, berberine has been studied most extensively [42,43,44]. In our studies, we focused on palmatine, whose activity against breast cancer has not been extensively investigated to the best of our knowledge. In the previously published work on the methanolic extract from Cretan barberry [41], the content of palmatine was calculated as 1.815 ± 0.21%. Palmatine was the fourth most abundant compound in the analyzed extracts after magnoflorine (7.078 ± 0.84%), berberine (5.382 ± 0.41%), and jatrorrhizine (2.2231 ± 0.09%), so its presence may influence the total activity of the extract to some extent. While analyzing the Scopus database, we found only 14 original papers that include the “palmatine” and “breast” and “cancer” topics. Among them, only one paper described a direct influence of palmatine on breast cancer cells in a photodynamic inactivation process [45], whereas the remaining publications showed studies on palmatine-containing extracts only.

The recent interest increase in palmatine is closely related to its inclusion in the monographs of European Pharmacopoeia. The number of records listing the studies on palmatine in the Scopus database increased significantly within the past 15 years. The total number of records mentioning palmatine in 2006 was equal to 26, whereas in 2020 this number was equal to 122.

In our studies, palmatine was isolated from natural sources using column chromatography. The application of Sephadex LH-20 resin provided high-purity palmatine for the study and was found to be a successful stationary phase for the barberry’s alkaloids separation in the mobile phase composed of methanol/water 50:50 (*v*/*v*). As many of the compounds are highly polar, silica gel remains insufficient for the separation of this group of secondary metabolites. Tailing can be observed together with strong absorption on the gel. The mechanism of separation on Sephadex columns enabled a complete elution of the dosed extract. Sephadex belongs to hydroxypropylated, crosslinked dextrans. Its particle size ranges from around 27 to 163 μm. It is supplied as a dry powder and must be swollen before use. The extent of resin swelling depends on the polarity of solvents used in the separation process. In this study, several solvent systems were tested to optimize the purification process of isoquinoline alkaloids—namely 100% dichloromethane, 50% dichloromethane in methanol, methanol, 50% methanol in water, and 40% methanol in water. The last two solvents were the most efficient for the separation of alkaloids, although the last one (MeOH/H_2_O, 40:60) resulted in too slow a separation. Consequently, we selected 50% aqueous methanol solution as the most effective one, as it provided a slow flow rate and satisfactory separation of the components. Previously, this technique was used for the purification of alkaloids from *Corydalis decumbens* by Huang and co-investigators [46] and from *Stephania yunnanensis* by Shi and collaborators [47].

We found that PLT at about 5 µg/mL markedly affected the viability and the proliferation of human ER^+^/HER2^−^ breast cancer cells (MCF7, T47D, and ZR-75-1), achieving 50% inhibition of cell viability. Our results differ from those of Wu et al. [45], where the half-maximal (50%) inhibitory concentration (IC_50_) of palmatine was obtained at 25 µM (9.6965 µg/mL), almost twice the dose we measured. However, the differences may be due to the shorter incubation period (24 h) with PLT in their case.

We have also shown that PLT was relatively selective towards breast cancer cells as determined by the Selectivity Index (SI). The SI is a crucial parameter used to determine the effectiveness of a herbal drug and/or isolated compound [48]. The SI of a compound is calculated by dividing the IC50 in normal cell line by the IC50 in the cancer cell line [49]. Usually, the higher the SI values, the higher selectivity of the studied compound or extract [50]. In our studies, the selectivity indexes of PLT ranged from 6.644 to 7.524, and the results were similar to those obtained in the studies done by Johnson-Ajinwo et.al. They showed that PLT is less cytotoxic to human ovarian epithelial (HOE) cells and that the selectivity indexes of PLT for ovarian cancer cell lines ranged from 3 to 5 [21]. Still, there are discrepancies in the interpretation of the minimum acceptable value of SI. For example, Oluyemisi et al., considered a SI ≤ 10 as weakly selective and a SI > 10 to be interesting in in vitro studies [51]. Meanwhile, a SI > 2 indicates selectivity and a SI = 5.5 indicates a good selectivity in another reports [52]. Moreover, Weerapreeyakul et al., proposed the a SI ≥ 3 for classifying a perspective anticancer sample [53]. Therefore, further research is needed to standardize the SI.

Promising anti-cancer activity of PLT has been also reported in in vivo models. A compound with the median lethal dose (LD_50_) between 500 and 5000 mg/kg is considered moderately toxic [54]. Acute toxicity studies on mice showed that the LD_50_ value of PLT was 1533.68 mg/kg. Moreover, there was no mortality and morbidity in rats in the subchronic toxicity studies after the oral administration of PLT [55]. Additionally, following 10 or 20 mg/kg/day oral PLT treatment, tumor numbers were significantly reduced in the small intestine and colon in mice [56]. These studies are consistent with a recent report [57] describing the anti-tumor effect of PLT in a xenograft model of colon cancer. It was shown that PLT could effectively induce regression of HCT-116 subcutaneous xenograft tumors without significant toxicity to the mice tested.

Based on available pharmacokinetics and pharmacodynamics profiles of berberine [58] and other protoberberine alkaloids, including PLT, the major limitation in the use of these compounds in clinic is their poor bioavailability, which is related to their low intestinal absorption and their efflux by permeability-glycoprotein (P-gp) [59]. One of the reported strategies for enhancing the efficacy of berberine as a promising anti-cancer drug involves its combination with other chemotherapeutic agents [60]. Combined treatment involving the use of drugs that work through a distinct mechanism is currently one of the most promising strategies for treatment activity in oncology which may reduce the likelihood of developing resistant cancer cells and prevent the occurrence of intolerable side effects associated with monotherapy [61]. There is growing evidence to suggest that certain natural products derived from plants, such as curcumin, xanthohumol, garcinol, genistein, luteolin, quercetin, mangiferin, furanodiene, and proanthocyanidins, enhance the effects of chemotherapy drugs in cancer treatment, including breast cancer [62]. The results we obtained exploring the efficacy of PLT in a combination of doxorubicin (DOX) against breast cancer cells obtained by us are novel. PLT sensitized breast cancer cells to DOX, and the mixture of PLT/DOX decreased cell viability more than either compound alone. Moreover, co-treatment of PLT with DOX generated the most desirable drug–drug interaction, such as a synergistic effect in ZR-75-1 cells and additive effects in MCF-7 and T47D cells, as revealed by the isobolographic analysis. Chakravarthy et al. [63] found that PLT also worked synergistically with gemcitabine to inhibit growth of pancreatic cancer cell lines. Many studies have reported a correlation between the inhibition of telomerase and the sensitization of cancer cells to chemotherapeutic agents [64]. An increased sensitivity of breast cancer cells to DOX indicated in our studies can result from the ability of PLT to induce and stabilize the G-quadruplex (G4) DNA structure in the telomere [65]. It has been shown that G4 structure is a poor substrate for telomerase [66] and that its formation leads to critical shortening of telomeric sequences. Consequently, it may result in the entry of cells into apoptosis, which is a desirable target in anti-cancer therapy [67,68].

To date, several targets and molecular mechanisms of antitumor action of PLT have been proposed. For example, PLT has the effect of inducing G2/M phase cell cycle arrest and mitochondrial-associated pathway apoptosis in HCT-116, HT-29, and SW-480 colon cancer cells by targeting kinase AURKA [57]. In prostate cancer cells, a possible target for PLT was receptor tyrosine kinase, such as S6 ribosomal protein (rpS6), a key regulator of protein synthesis and a downstream effector of the Akt/mTOR/p70S6K signaling pathway. Further, it has been found that PLT inhibited the invasion of DU-145 prostate cancer cells, which was accompanied by reduced transcriptional activity of *NF-κB* and subsequently its downstream target gene *c-FLIP* [24]. The ability of PLT to suppress proliferation and invasion has also been tested against pancreatic cancer cells. PMT-mediated increased apoptosis was reflected by the decrease in expression and levels of survivin and in the induction of PARP cleavage [63]. As we have mentioned, the studies of PLT in breast cancer cells are limited. Interestingly, in the case of triple-negative breast cancer cell lines, we found that the potential of PLT to inhibit cell survival/proliferation was also promising, or even comparable to that obtained for the studied estrogen receptor-positive breast cancer cell lines (data not shown). It suggests that the cytotoxic activity of PLT may not be mediated through hormone-dependent signaling pathways, and further studies need to be conducted.

## 4. Materials and Methods

### 4.1. Plant Material

The roots of *Berberis cretica* used for the isolation of palmatine for the purpose of this study were collected by Eleftherios Kalpoutzakis on the island of Crete (Greece), from the rocky locations of the Rouvas forest on the Nidha Plateau, where it grows in the vicinity of *Quercus coccifera* trees in the fall. The plant material belongs to the herbarium of Alexios Leandros Skaltsounis’s research group in the Department of Pharmacognosy and Natural Compounds Chemistry at the University of Athens, Greece and was given a voucher specimen number: KL006R. The roots were finely cut, air-dried and ground prior to the extraction.

### 4.2. Extraction

Fifty grams of pulverized roots of *Berberis cretica* were extracted in a stainless steel vessel of ASE extractor (accelerated solvent extractor, ASE 100, Dionex, Sunnyvale, CA, USA) using methanol as an extracting solvent applying flowing parameters: extraction time = 10 min, number of extraction cycles = 3, temperature = 80 °C, purge time = 60 s [27]. The obtained extract was evaporated at 45 °C on a rotary evaporator and subjected to fractionation.

### 4.3. Isolation of Palmatine from Root Extract

Methanolic extract from the roots of *Berberis cretica* (5 g) was redissolved in 10 mL of methanol and was dried again on a rotary evaporator at 45 °C together with 5 g of normal phase silica gel 60H (Merck, Darmstadt, Germany). The obtained powder was transferred to the top of a Buchner separating glass funnel filled with normal phase silica gel. The extract was fractionated using 100 mL of the following solvents, each: dichloromethane and 10%, 20%, 50%, and 100% methanol in dichloromethane that was removed with the help of vacuum. The composition of collected fractions was analyzed using TLC chromatography, pooled when similar, and evaporated to dryness. The fraction obtained with 20% of methanol (520 mg) in dichloromethane was further fractionated on a glass column filled with Sephadex LH-20 resin (Sigma-Aldrich, St. Louis, MO, USA). Soaked for 2 h Sephadex LH-20 beads were moved into a glass column in one continuous motion. When the level of solvent reached the top of the resin level, the extract dissolved in methanol: water (50:50 *v*/*v*) was loaded on a column, and the elution proceeded in a flow rate of around 10cm/h. As a mobile phase methanol/water (50:50 *v*/*v*) mixture was used for the first 90 fractions and methanol for the remaining 42 fractions, yielding 48 mg of palmatine.

The purity of the isolate was analyzed by the HPLC-DAD chromatograph (Shimadzu, Kyoto, Japan) composed of a degasser (DGU-20A3R), a quaternary pump (LC-20AD), an autosampler (SIL-20AHT), and a PDA detector (SPD-M20A) on the Sigma-Aldrich (St. Louis, CA, USA) Discovery RP-18 chromatographic column (d = 5.0μm, 250.0 mm × 4.0 mm). The following gradient of water with an addition of 2% of glacial acetic acid (J.T. Baker) (A) and acetonitrile with 2% acetic acid (B) was applied: 0 min, 0.5% B; 40 min, 20% B; 60 min, 40% B; 70 min, 0.5% B. The method’s length was set at 70 min, the post run at 4 min, the flow rate at 1 mL/min, the temperature at 25 °C, and the detection wavelengths at 260, 280, and 365 nm.

The final identification of the isolate was performed by an HPLC-ESI-QTOF-MS/MS platform that was composed of the HP1200 Series Agilent Technologies (Santa Clara, CA, USA) chromatograph with QTOF-MS/MS (6500 Series) detector in a quick gradient of solvent A (0.2% formic acid) and solvent B (0.2% formic acid in acetonitrile): 0 min 10% of A in B; 10min 95% of A in B; 13 min 95% of A in B;14 min 10% of A in B. The separation was performed on the chromatographic column Zorbax Eclipse Plus Stablebond RP-18 (150 × 2.1 mm, 3.5 µm) by Agilent Technologies (Santa Clara, CA, USA) using the previously described instrumentation [27]. The major mass spectrometer operational settings included drying and sheath gas temperatures of 325 and 350 °C, a capillary voltage of 3500 V, a gas flow rate of 12 L/min, fragmentation voltage of 130 V, the collision (CID) energies of 10 and 20 V, and skimmer voltage of 65V. The identity of palmatine was confirmed by the analysis of its MS/MS spectra and a comparison with the previously recorded data in the authors’ library, with the scientific literature, and with open access databases of mass spectrometry data (Metlin).

### 4.4. Cell Lines

Breast cancer cell lines belonging to the ER^+^/PR^+^/HER2^−^ subtype such as MCF-7 (HTB-22™), T47D (HTB-133™) and ZR-75-1 (CRL-1500™) [69,70] and normal human breast epithelial cells, MCF-10A (CRL-10317™), were obtained from the American Type Culture Collection (ATCC). T47D and ZR-75-1 cell lines were maintained in RPMI 1640 medium (Sigma Chemicals, St. Louis, MO, USA). MCF-7 was grown in Eagle’s minimal essential medium (EMEM) (ATCC) with 0.01 mg/mL human recombinant insulin (Sigma Chemi-cals). Nutrient mixture F-12 Ham culture medium (Sigma Chemicals) with 20 ng/mL human epidermal growth factor (hEGF) (Sigma Chemicals), 10 µg/mL bovine insulin (Sigma Chemicals) and 500 ng/mL hydrocortisone (Sigma Chemicals) was used for culture of MCF-10A cells. All culture media were supplemented with 10% fetal bovine serum (FBS) (Sigma Chemicals), penicillin (100 µg/mL) (Sigma Chemicals) and streptomycin (100 µg/mL) (Sigma Chemicals). Cultures were kept at 37 °C in a humidified atmosphere of 95% air and 5% CO_2_.

### 4.5. Cell Viability Assessment

Cell viability was measured using the MTT method, based on the ability of the mitochondria of viable cells to reduce yellow tetrazolium salt (MTT) to purple formazan crystals. MCF-7, ZR-75-1, and T47D cells were seeded in 96-well plates (Nunc, Roskilde, Denmark) at a density of 1 × 10^5^ cells/mL, 5 × 10^4^ cells/mL, and 2 × 10^4^ cells/mL, respectively. Next, attachment cells were treated with increasing concentrations of palmatine and doxorubicin for 72 h. Following, cells were incubated with MTT solution (5 mg/mL, Sigma Chemicals) for 3 h at 37 °C. The absorbance was measured at 570 nm with an Infinite M200 Pro microplate reader (Tecan, Männedorf, Switzerland) after adding sodium dodecyl sulfate (SDS) buffer (10% SDS in 0.01 N HCl) for overnight.

### 4.6. Cell Proliferation Assay

Cell Proliferation ELISA, BrdU Kit (Roche Diagnostics, Mannheim, Germany) was used following manufacturer’s instructions. Optimized amounts of MCF-7 (1 × 10^5^/mL), ZR-75-1 (5 × 10^4^/mL), and T47D (2 × 10^4^/mL) cells were placed on a 96-well plate (Nunc) (100 μL/well). On the next day, the cancer cells were treated with increased concentrations of palmatine for 48 h, followed by 10 µL/well BrdU Labeling Solution (100 µM) which was added, and cells were reincubated for an additional 24 h at 37 °C. Then, the culture medium was removed and cells were fixed in FixDenat solution (200 µL/well) (30 min, room temperature (RT)). The working solution of anti-BrdU antibody coupled with horseradish peroxidase (anti-BrdU-POD) was subsequently added (100 µL/well) (90 min, RT) and detected using tetramethylobenzidine substrate (TMB) (100 µL/well) (30 min, RT). An amount of 1 M sulfuric acid was added (25 µL/well) to stop enzymatic reaction, and quantitation was performed spectrophotometrically at 450 nm using an Infinite M200 Pro microplate reader (Tecan).

### 4.7. Cytotoxicity Assessment—LDH Assay

MCF-10A, MCF7, T47D, and ZR-75-1 cells were plated at a seeding density of 1 × 10^5^ cells/mL, 1 × 10^5^/mL, 2 × 10^4^/mL, and 5 × 10^4^/mL, respectively, on 96-well plates (Nunc) and incubated at 37 °C for 24 h. Then, cells were washed with PBS, and various concentrations of palmatine were applied to the cells. The cytotoxicity was estimated based on the measurement of cytoplasmic lactate dehydrogenase (LDH) activity released from damaged cells after 72 h exposure to palmatine. LDH assay was performed according to manufacturer’s instruction (Cytotoxicity Detection KitPLUS LDH) (Roche). Triton X-100 solution was used as positive control to measure the maximum releasable LDH in the cells. The average values of the culture medium background were subtracted from all values of experimental wells, and the percentage of dead cells was calculated in relation to the maximum LDH release.

### 4.8. Apoptosis Detection

The assay was conducted using Annexin V/propidium iodide staining and a NucleoCounter NC-3000 (ChemoMetec, Allerod, Denmark). The cells were seeded into 6-well plates and were incubated for 72 h with PLT or without as a control. Next, cells were detached from the plate using Trypsin–EDTA solution (Corning, Corning, NY, USA) and were twice resuspended in PBS. After the centrifugation of cell solutions at 400× *g* at room temperature, the obtained cell pellets were resuspended in 100 μL of Annexin V binding buffer. Then, cells were stained using 2 μL of Annexin V–CF488A conjugate and 4 μL of Hoechst 33,342 (10 μg/mL), respectively. The samples were incubated for 15 min at 37 °C, centrifuged at 400× *g* for 5 min, and were resuspended in 100 μL of Annexin V binding buffer supplemented with 10 μg/mL PI (prepared by adding 2 μL Solution 16 to 100 μL binding buffer). The suspension of stained cells was loaded into the NC-slide and analyzed in a NucleoCounter NC-3000. Each experiment was conducted three times, with measurements in triplicate.

### 4.9. Isobolographic Analysis of Interactions

Isobolographic analysis is a statistical method allowing the characterization of pharmacodynamic interaction between drugs. Initially carried out, log-probit analysis, according to Litchfield and Wilcoxon [30], was used to determine the percentage of inhibition (also median inhibitory concentrations IC_50_) of cell viability per dose of DOX and PLT when administered singly in the MCF-7, T47D, and ZR-75-1 cell lines measured in vitro by the MTT assay. The test for parallelism of concentration–response effect lines for PLT and DOX was performed. This has an impact on the appearance of the additivity line on the isobologram. [71,72,73]. Interactions between PLT and DOX in studied breast cancer cell lines were isobolographically analyzed, as described elsewhere [71,74,75,76]. The median additive inhibitory concentrations (IC_50 add_) for the mixture of PLT with DOX, which theoretically should inhibit 50% of cell viability, were calculated, as demonstrated by Tallarida [75,76]. The assessment of the experimentally derived IC_50 exp_ at the fixed-ratio of 1:1 was based on the concentration of the mixture of PLT and DOX of cell viability in cancer cell lines measured in vitro by the MTT assay. Details concerning the isobolographic analysis have been published elsewhere [71,75,76].

### 4.10. Statistical Analysis

Statistical analysis was performed using GraphPad Prism 6 statistical software. One-way analysis of variance (ANOVA test) with Tukey’s significance test was used for multiple comparisons. Results are expressed as the mean ±standard error (SEM) (* *p* < 0.05, ** *p* < 0.01, *** *p* < 0.001, **** *p* < 0.0001). The IC_50_ and IC_50 exp_ values for PLT and DOX administered alone or in combination at the fixed-ratio of 1:1 were calculated by computer-assisted log-probit analysis according to Litchfield and Wilcoxon [30]. The experimentally-derived IC_50 exp_ values for the mixture of PLT with DOX were statistically compared with their respective theoretical additive IC_50 add_ values by the use of unpaired Student’s *t*-test, according to Tallarida [77].

## 5. Conclusions

The present study showed that PLT, isolated from *Berberis cretica* methanolic root extract by column chromatography, significantly inhibited the growth of human estrogen receptor-positive breast cancer cell lines in vitro. Thanks to the analysis of several breast cancer cells, we clearly demonstrated that the anti-cancer activity of PLT is not specific to the cell line and can be generalized for tumor type. Moreover, our findings revealed, for the first time, the lower toxicity of PLT against MCF10A normal human breast epithelial cells in comparison to the tumor cell lines, which was confirmed by two independent assays, MTT and LDH. In addition, this is the first report indicating the anti-proliferative activity of PLT in combination with DOX against breast cancer cells. Isobolographic analysis demonstrated that the most desired, synergistic interaction, was revealed between PLT and DOX in ZR-75-1 cells. Additive effects between studied agents, which can also be advantageous, have been found in MCF and T47D cell lines.The results obtained in our studies suggest that PLT can be a potential candidate agent for preventing and treating breast cancer. However, further intensive investigations are required to provide the possible mechanism action of PLT either alone or in combination with DOX.

## Figures and Tables

**Table 1:** The anti-proliferative effects of palmatine (PLT) and doxorubicin (DOX) administered singly in human breast cancer cell lines measured in vitro by the MTT assay. Data are median inhibitory concentrations (IC_50_ values in μg/mL ± SEM) of PLT and DOX administered separately in cancer cell lines.

Cell Line	Drug	IC_50_ µg/mL
MCF7	PLT	5.194 ± 1.492
	DOX	0.104 ± 0.029
T47D	PLT	5.805 ± 2.048
	DOX	0.089 ± 0.020
ZR-75-1	PLT	5.126 ± 1.774
	DOX	0.080 ± 0.028

## Data Availability

Data is contained within the article.

## Sample Availability

Samples of the compounds such as palmatine and doxorubicine are available from the authors.
